# Patterns of Surgically Managed Maxillofacial Injuries in a Tertiary Care Hospital in North India

**DOI:** 10.7759/cureus.112470

**Published:** 2026-07-11

**Authors:** Armaan Khosa, Vinay Karwal

**Affiliations:** 1 Plastic Surgery, Adesh Medical College and Hospital, Shahbad, IND

**Keywords:** craniomaxillofacial surgery, facial trauma, maxillofacial injuries, open reduction internal fixation, panfacial fracture

## Abstract

Objectives: Maxillofacial injuries represent a significant burden in trauma care, largely driven by the increasing incidence of motor vehicle collisions. This study evaluates the demographic profiles, aetiologies, fracture patterns, concomitant injuries, and clinical outcomes of surgically managed maxillofacial trauma at a tertiary care centre in North India.

Methods: This retrospective observational study analysed patients aged >15 years undergoing surgical intervention for acute maxillofacial fractures between January and December 2024. Data regarding demographics, mechanism of injury, fracture site, associated trauma, and postoperative complications were extracted from hospital records and analysed using IBM SPSS Statistics for Windows, Version 27 (Released 2020; IBM Corp., Armonk, New York).

Results: Of the 152 patients, 96.05% (n = 146) were male, with the most prevalent age group being 21-30 years (26.32%). Road traffic accidents (RTAs) were the primary aetiology (80.26%). Panfacial fractures were the most common injury pattern (53.95%). Concomitant head injuries were present in 36.84% of cases. The average hospital stay was 5.6 days, with aesthetically unpleasing scars being the most frequent postoperative concern.

Conclusion: The high prevalence of RTA-induced panfacial fractures among young males highlights an urgent need for stricter enforcement of road safety protocols, speed regulations, and the use of protective gear in the North Indian region.

## Introduction

The face is highly susceptible to injuries, which can affect the bones, teeth, or soft tissues [1]. Most maxillofacial fractures can be categorised as resulting from road traffic accidents (RTAs), falls, or physical assaults [2]. According to statistics from the World Health Organisation (WHO), approximately 25% of all fatalities due to injuries are attributed to RTAs [3]. Facial trauma can lead to several cosmetic and functional problems. While many fractures are managed conservatively, surgically treated cases represent the most severe end of the trauma spectrum, requiring significant resource allocation. This study aims to examine the different patterns of surgically managed maxillofacial injuries in a tertiary care hospital, with respect to demographic factors, causes, fracture types and locations, treatment approaches, and clinical outcomes.

## Materials and methods

This hospital-based retrospective observational study was conducted in the Department of Plastic and Reconstructive Surgery at a tertiary care hospital in North India. The study protocol was reviewed by the institutional ethics committee, and approval was obtained for carrying out the study. All patients presenting to the hospital with acute maxillofacial fractures from January 1, 2024, to December 31, 2024, who were surgically managed, were included in the study.

Inclusion criteria

All patients aged >15 years presenting with acute maxillofacial fractures who underwent surgery were included.

Exclusion criteria

Patients presenting >21 days post-injury (delayed presentation) were excluded to ensure a focus on acute trauma management. Patients with soft tissue injuries alone and no fractures, patients managed conservatively, and those with life-threatening concomitant injuries were excluded from the study.

After approval from the administration, data were collected from hospital records, admission sheets, operative notes, and discharge summaries. A structured data collection form was used, which included demographics (age, sex), mechanism of injury, types of fractures, concomitant injuries, and complications or outcomes at discharge (Appendices). The variables were entered into Microsoft Excel and analysed using IBM SPSS Statistics for Windows, Version 27 (Released 2020; IBM Corp., Armonk, New York). Qualitative data are presented as frequencies, whereas quantitative data are presented as means.

## Results

In 2024, the emergency department of our hospital received a total of 1457 trauma cases. A total of 152 patients met the inclusion criteria from an initial pool of 246 maxillofacial trauma presentations. Of these, 146 were male (96.05%) and 6 were female (3.95%), resulting in a stark male-to-female ratio of 24:1. Patients were aged 17-61 years, with most in the 21-30 years age group (26.32%). Two-thirds of the patients (65.79%) were under 40 years of age (Table 1).

**Table 1:** Age distribution of maxillofacial injury patients The table shows the number and percentage of cases of maxillofacial injuries for each age group (years).

Age group (years)	Number of cases	Percentage of cases (%)
≤20	28	18.42
21-30	40	26.32
31-40	32	21.05
41-50	32	21.05
51-60	16	10.53
61-70	4	2.63

A total of 122 cases (80.26%) were due to RTAs, the most common aetiology of maxillofacial injuries in our study. Other causes included physical assaults, falls, and sports injuries (Table 2). 

**Table 2:** Mechanisms of injury The table shows the number and percentage of maxillofacial injury cases by mechanism.

Mechanism of injury	Number of cases	Percentage of cases (%)
Road traffic accidents	122	80.26
Physical assault	16	10.53
Falls	10	6.58
Sports injuries	4	2.63

Various anatomical locations and fracture patterns were observed in the study. These included panfacial fractures, frontal bone fractures, mid-face fractures, and mandible fractures. Mid-face fractures included zygomatic fractures, maxillary fractures, nasal bone fractures, and orbital fractures (Table 3). 

**Table 3:** Distribution of fracture patterns The table shows the number and percentage of cases of maxillofacial injuries with the locations of the fractures. ZMC: zygomaticomaxillary complex.

Location of fracture	Number of cases	Percentage of cases (%)
Panfacial	82	53.95
Upper face (frontal bone)	4	2.63
Mid-face (ZMC)	28	18.42
Lower face (mandible)	38	25

Maxillofacial trauma was associated with internal head injuries in 56 cases (36.84%). Other concomitant injuries included trauma to the limbs, chest, abdomen, and spine (Table 4).

**Table 4:** Concomitant injuries with maxillofacial injuries The table shows the number and percentage of cases of maxillofacial injuries with associated injuries.

Type of concomitant injury	Number of cases	Percentage of cases (%)
Head	56	36.84
Limb	14	9.21
Chest	20	13.16
Abdomen	8	5.26
Spine	6	3.95

The average hospital stay was 5.6 days, ranging from three to 15 days. The hospital stay was longer in patients with panfacial fractures and in those with other associated injuries in addition to the maxillofacial trauma. The most common complication in our study was unpleasant facial scars due to lacerations sustained at the time of trauma. Others included contour deformities and malocclusion. All 152 patients were successfully managed and discharged alive. All patients were on regular follow-up for a period of six months. Table 5 outlines the specific patient outcomes and clinical complications observed at the time of discharge. 

**Table 5:** Patient outcomes and complications at discharge (N = 152)

Status/outcome measure	Number of cases	Percentage of cases (%)
Successful discharge (survival)	152	100.00%
Unpleasant facial scars	14	9.21%
Contour deformities	11	7.24%
Malocclusion (misaligned teeth)	2	1.32%
Bone malunion (improper healing)	0	0.00%
Bone nonunion (unhealed bones)	0	0.00%
Osteomyelitis (bone infection)	0	0.00%
Enophthalmos (sunken eye)	0	0.00%

## Discussion

The incidence of maxillofacial injuries is steadily on the rise due to an increase in traffic, and the lack of preventive strategies in road traffic contributes to accidents, which are a primary cause of maxillofacial fractures [4]. The causes of maxillofacial fractures can be generally classified as RTAs, falls, assaults, sports-related incidents, industrial injuries, and injuries from warfare [5].

According to the World Health Organisation, annually, 1.35 million individuals lose their lives, and 15 to 30 million sustain injuries from RTAs [6]. The increasing incidence of RTAs in developing nations may be attributed to insufficient awareness regarding road safety practices, a negligent attitude among road users towards following safety protocols, inadequate road conditions, breaches of speed limits, and substandard driving skills. There is a sharp increase in the number of two-wheelers on the roads, coupled with flawed intersection design, speed bumps, narrow roadways, and heavy traffic, all of which are leading to an increase in accidents. The coexistence of fast- and slow-moving vehicles in the same lane, disregard for protective gear, and violations of highway regulations are additional factors that hamper road safety. Using a car with airbags and ensuring that all passengers wear seat belts can reduce injuries from RTAs [7]. The high prevalence of RTAs as the primary aetiology (80.26%) reflects a trend common in developing nations. Unlike Western countries, where interpersonal violence or sports injuries often lead the statistics, our findings align with studies from other Indian tertiary centres where high-velocity vehicular trauma predominates. This is largely due to rapid urbanisation, a high volume of two-wheelers, and inconsistent use of safety gear such as helmets and seat belts.

There is a noticeable male predominance in maxillofacial injuries, with the male-to-female ratios for facial trauma resulting from road accidents being as high as 7.6:1 in one of the studies [8]. Injuries from falls and sports primarily affect younger individuals, as this demographic tends to be more active and engaged in sports and other extracurricular activities. Assaults and RTAs predominantly occur among people aged 20 to 40 years [8]. Our demographic data show a profound male predilection (96.05%), with a peak incidence in the 21-30-year age group. This demographic represents the most mobile and economically active segment of the population, often engaging in high-risk driving behaviours. The low incidence among females (3.95%) may be attributed to lower exposure to outdoor professional activities and driving in this specific geographic region.

Computed tomography (CT) is the key diagnostic tool for assessing maxillofacial fractures because it can accurately detect even small fractures and their associated complications, aiding surgical planning [9]. All patients with maxillofacial injuries at our trauma centre undergo a non-contrast CT scan of the face, consisting of coronal and axial cuts with three-dimensional reconstruction.

Facial fractures can be classified in terms of their anatomical location as frontal (upper one-third), mid-face (middle one-third), mandibular (lower one-third), and panfacial fractures. Panfacial fractures are those involving bones of more than one region of the face. Mid-face fractures include zygomatic, maxillary, nasal, and orbital fractures. Mid-face fractures can also be classified as Le Fort fractures, involving fractures of the pterygoid plates.

Mid-face fractures accounted for 41% of cases, while mandibular fractures accounted for 39% in the research conducted by Agrawal et al. [10]. This study indicated a statistically significant rise in the occurrence of mid-face fractures and a rising trend in panfacial fractures, which may be related to the changing aetiology, as high-speed two-wheeler trauma is now more dominant. This observation slightly contrasts with findings from other studies [7]. Chandra et al. reported isolated mandibular fractures in as many as 48.6% of cases in their study, with mid-face fractures following at 27.6% [11]. The high rate of panfacial fractures in our study, 53.95%, is a critical finding, indicating high-energy impacts. Our hospital, being located on the national highway and serving as the regional trauma centre, receives a large number of RTA cases each day. The correlation between these fractures and concomitant head injuries (36.84%) underscores the necessity of a multidisciplinary approach involving neurosurgeons and plastic surgeons. The average hospital stay of 5.6 days was significantly influenced by the presence of associated injuries. Concomitant injuries, including head injuries, limb injuries, and thoracoabdominal injuries, along with maxillofacial trauma, were reported in 62.52% of the cases in the study by Agrawal et al., with the most common being head injury in 29.64% of cases [10]. Patil et al. documented associated injuries with maxillofacial trauma in 54% of patients in their study, with the most frequent associated injury being head injury (60%) [12].

Maxillofacial fractures can be managed either conservatively or operatively. Conservative management involves jaw rest, a liquid diet, and anti-oedema measures such as anti-inflammatory medication, head elevation, and ice fomentation. Indications for operative management include deranged occlusion, reduced mouth opening, diplopia, restriction in eye movements, enophthalmos or orbital dystopia, and deformities or a deranged facial contour.

Operative intervention improves function and optimises the cosmetic appearance of the face. All patients in this study underwent surgery under general anaesthesia, with nasal intubation being the preferred route of intubation. Various types of surgery were performed in this study. Open reduction and internal fixation with mini-plates and screws were performed for fractures of the mandible, zygomaticomaxillary complex, orbits, nasoethmoidal bones, and frontal bone. Closed reduction of the nasal bone and septum was performed in cases of the respective fractures, along with splinting. Closed reduction with arch bar fixation and intermaxillary fixation was performed for condylar fractures of the mandible.

Most of the patients required open reduction and internal fixation (ORIF) for their facial fractures in the research by Agrawal et al. [10]. Only 4.78% of the total 1046 cases were managed conservatively with a soft diet, rest, and anti-oedema measures.

All our patients were successfully discharged at the end of treatment. Common complications arising from maxillofacial injuries are facial scars, contour deformities, malocclusion, malunion, non-union, osteomyelitis, and enophthalmos [13]. These can be prevented by choosing the right approach to treatment. While malocclusion and non-union were rare in our study, unpleasant facial scarring was the most common patient concern. Most of these unpleasant scars were due to severe facial trauma with deep lacerations. There were some cases of contour deformities, which were attributed to facial tissue atrophy, tissue loss, and scar contraction. These too occurred in cases of severe facial trauma. The severity of condylar fractures can affect the outcome of closed reduction. One of our patients with a high condylar fracture of the mandible, who later had malocclusion, was not compliant with intermaxillary fixation. He later underwent orthodontic treatment. This highlights that while functional restoration is the primary surgical goal, the aesthetic outcome remains a major factor in patient satisfaction and psychological recovery following trauma.

The introduction of compulsory seat belt legislation has led to a reduction in the severity of trauma in motor vehicle accidents (MVAs) over the past decades [14]. The incidence of MVAs is also reduced in regions that enforce speed limits. Another example of the impact of legislative change on health is the effect of lowering the legal drinking age, as alcohol is a major contributing factor [15]. Alcohol intake affects judgement. These examples show how law enforcement can reduce the incidence of MVAs. The role of paramedical staff in transferring patients from the accident site to the hospital is of utmost importance, as they must be well-versed in primary care protocols [12]. Hence, paramedical staff should be thoroughly trained in emergency management protocols.

Regular audit of trauma data is important in planning hospital workload, for government authorities to determine funding allocations, and for healthcare providers to provide education and training in specific skills [16]. It also helps revise treatment algorithms for patients and enables relevant organisations to direct education towards preventing such trauma.

Our finding that RTAs account for over 80% of injuries aligns with studies from other developing economies but is significantly higher than reports from Western Europe, where interpersonal violence often predominates. The high incidence of panfacial fractures (53.95%) in our study suggests high-velocity impact, likely due to a lack of helmet use and seat belt compliance, as evidenced by the high rate of associated head injuries (36.84%).

This study has some limitations. One limitation could be discrepancies in the data, as they were collected from hospital records. Another is the short duration of the study. A longer study period would be required to ascertain trends in maxillofacial trauma. The study included only surgically managed patients. As a retrospective study from a single tertiary care centre, these results may reflect regional trauma patterns and might not be generalisable to all of India. Future prospective multicentre studies are recommended to help formulate national safety policies.

## Conclusions

To conclude, maxillofacial trauma has become increasingly common, particularly among younger age groups and in association with motor vehicle accidents. Panfacial fractures were the most common pattern observed, frequently accompanied by concomitant head injuries. This high burden of polytrauma is attributable to factors such as high-velocity impact and poor compliance with seat belt and helmet use. Timely surgical intervention plays a crucial role in reducing the morbidity and mortality associated with facial injuries. Equally important is the enforcement of road safety measures, with traffic authorities ensuring stricter compliance, an approach that could substantially reduce the incidence and severity of these injuries.

## Figures and Tables

**Figure 1:**
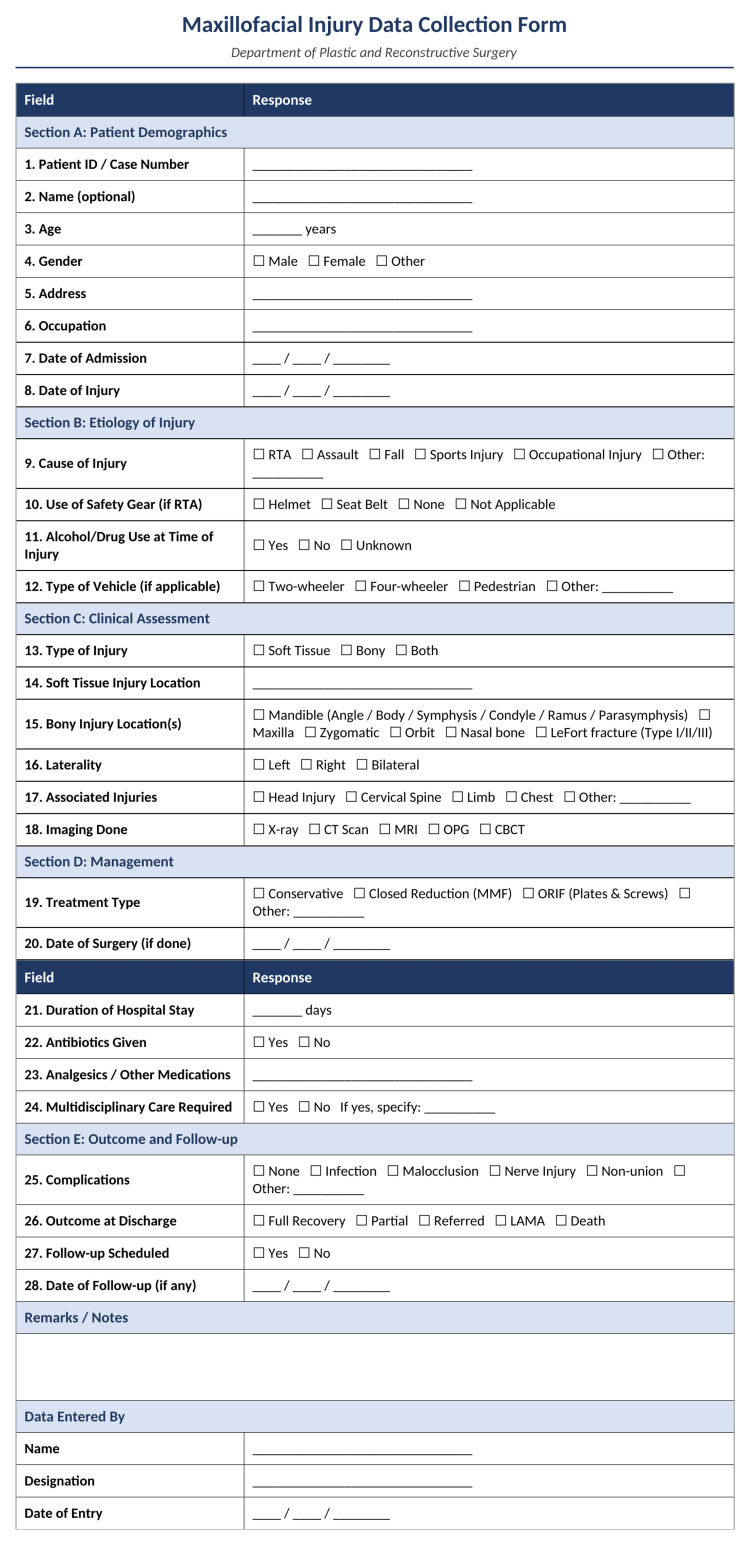
Maxillofacial injury data collection form
